# TGF-beta signal transduction: biology, function and therapy for diseases

**DOI:** 10.1186/s43556-022-00109-9

**Published:** 2022-12-19

**Authors:** Yan Tie, Fan Tang, Dandan Peng, Ye Zhang, Huashan Shi

**Affiliations:** 1 Department of Biotherapy, State Key Laboratory of Biotherapy and Cancer Center, West China Hospital, Sichuan University, No.37 Guo Xue Xiang, Chengdu, 610041 China; 2 Orthopaedic Research Institute, Department of Orthopaedics, West China Hospital, Sichuan University, Chengdu, China; 3 Department of Radiation Oncology, National Cancer Center/National Clinical Research Center for Cancer/Cancer Hospital, Chinese Academy of Medical Sciences and Peking Union Medical College, Beijing, 100021 China

**Keywords:** TGF-beta, Targeted therapy, Tumor microenvironment, Fibrosis, Anemia

## Abstract

The transforming growth factor beta (TGF-β) is a crucial cytokine that get increasing concern in recent years to treat human diseases. This signal controls multiple cellular responses during embryonic development and tissue homeostasis through canonical and/or noncanonical signaling pathways. Dysregulated TGF-β signal plays an essential role in contributing to fibrosis via promoting the extracellular matrix deposition, and tumor progression via inducing the epithelial-to-mesenchymal transition, immunosuppression, and neovascularization at the advanced stage of cancer. Besides, the dysregulation of TGF-beta signal also involves in other human diseases including anemia, inflammatory disease, wound healing and cardiovascular disease et al. Therefore, this signal is proposed to be a promising therapeutic target in these diseases. Recently, multiple strategies targeting TGF-β signals including neutralizing antibodies, ligand traps, small-molecule receptor kinase inhibitors targeting ligand–receptor signaling pathways, antisense oligonucleotides to disrupt the production of TGF-β at the transcriptional level, and vaccine are under evaluation of safety and efficacy for the forementioned diseases in clinical trials. Here, in this review, we firstly summarized the biology and function of TGF-β in physiological and pathological conditions, elaborated TGF-β associated signal transduction. And then, we analyzed the current advances in preclinical studies and clinical strategies targeting TGF-β signal transduction to treat diseases.

## Introduction

Cytokine-based targeting presents a promising therapy in many disorders, including cancer, inflammatory or infectious diseases, and fibrotic diseases [1–3]. One multifunctional polypeptide cytokine, transforming growth factor beta (TGF-β), becomes a potential therapeutic target for its bio function in regulating the growth and differentiation of cells. This cytokine belongs to the TGF-β superfamily, which comprises many proteins, including growth differentiation factors and activins. The structurally-related TGF-β1, TGF-β2, and TGF-β3 cytokines are three isoforms of the TGF-β family [4, 5]. If not otherwise specified, TGF-β in the following statement will stand for TGF-β1. Generally, latent TGF-β is stored in the multiple extracellular matrix. Under enzymatic and non-enzymatic action, latent TGF transforms into activated TGF. Only activated TGF can bind to the TGF receptor complex and induce canonical and noncanonical pathways of TGF-β signal transduction.

Although scientists have proposed the critical role of TGF-β signaling pathways in fibrosis, tumorigenesis, and regulating immune responses, developing TGF-β-targeted therapeutic drugs is a great obstacle for the dual role and paradoxical effects on fibrosis and immune systems regulation in the occurrence and development of disease. TGF-β is critical in regulating tissue homeostasis and renewal in physiological conditions. In pathological conditions, TGF-β signaling plays a critical role in regulating inflammatory progression and wound healing [6–8]. Moreover, TGF-β signaling also contributes to fibrosis by inducing extracellular matrix deposition [4, 9]. Dysregulated TGF-β regulates both adaptive immunity and innate immunity during tumorigenesis. At early tumorigenesis, TGF-β becomes cytostatic, apoptotic, and tumor suppressive and acts as a tumor suppressor by inhibiting excessive inflammation and inducing tolerance. While during advanced cancer, TGF-β is necessary to promote tumor tolerance, inflammation suppression, T cell exclusion, epithelial-mesenchymal transition, migration, invasion, and progression [10].

Regulation of TGF-β signal transduction occurs at several levels, including the production of TGF-β ligands, ligand-receptor interactions, downstream signal cascades after kinase receptor activation, and transcriptional disruption. These crucial roles and promising therapeutic potential of TGF-β in the diseases mentioned above, the specific mechanisms of TGF-β driving these diseases and therapies based on TGF-β signal transduction provide targeted therapeutic strategies. Recently, many TGF-β-targeted drugs are under preclinical and clinical trials. Neutralizing antibodies, TGF-β ligand traps, small-molecule receptor kinase inhibitors, antisense oligonucleotides, and vaccine-based therapy are the main targeted strategies of TGF-β [11–15]. However, most of them are in phase 1/2 clinical trials. In this review, we elaborated on the biology and function of TGF-β, and summarized the recent advances in TGF-β associated targeted therapy.

## The biology of TGF-β

### The production and activation of TGF-β

TGF-β usually exists extracellularly as heterodimers or homodimers [16]. Generally, TGF-βs have three mammalian genome-encoded isoforms: TGF-β1, TGF-β2, and TGF-β3 [4]. Each isoform is synthesized in the rough endoplasmic reticulum as a precursor molecule that consists of an N-terminal signal peptide, the latency-associated polypeptide (LAP), and a mature polypeptide at the C-terminal [17]. When the signal peptide is removed, the precursor is elaborated through proteolytic cleavage, thereby separating the N-terminal prodomain from the C-terminal mature polypeptide. Among the three isoforms, the TGF-β1 homodimer is the most widely studied subtype and was the first purified protein, which is characterized by complementary DNA cloning [18]. For cell origination, TGF-β1 is originally purified from platelets [19]. In addition, tumor cells, tumor-associated macrophages and stromal cancer-associated fibroblasts in the tumor microenvironment also express TGF-β1 in a heterogeneous manner, not necessarily the expression of TGF-β2 or TGF-β3 [17, 20, 21].

**Fig. 1:**
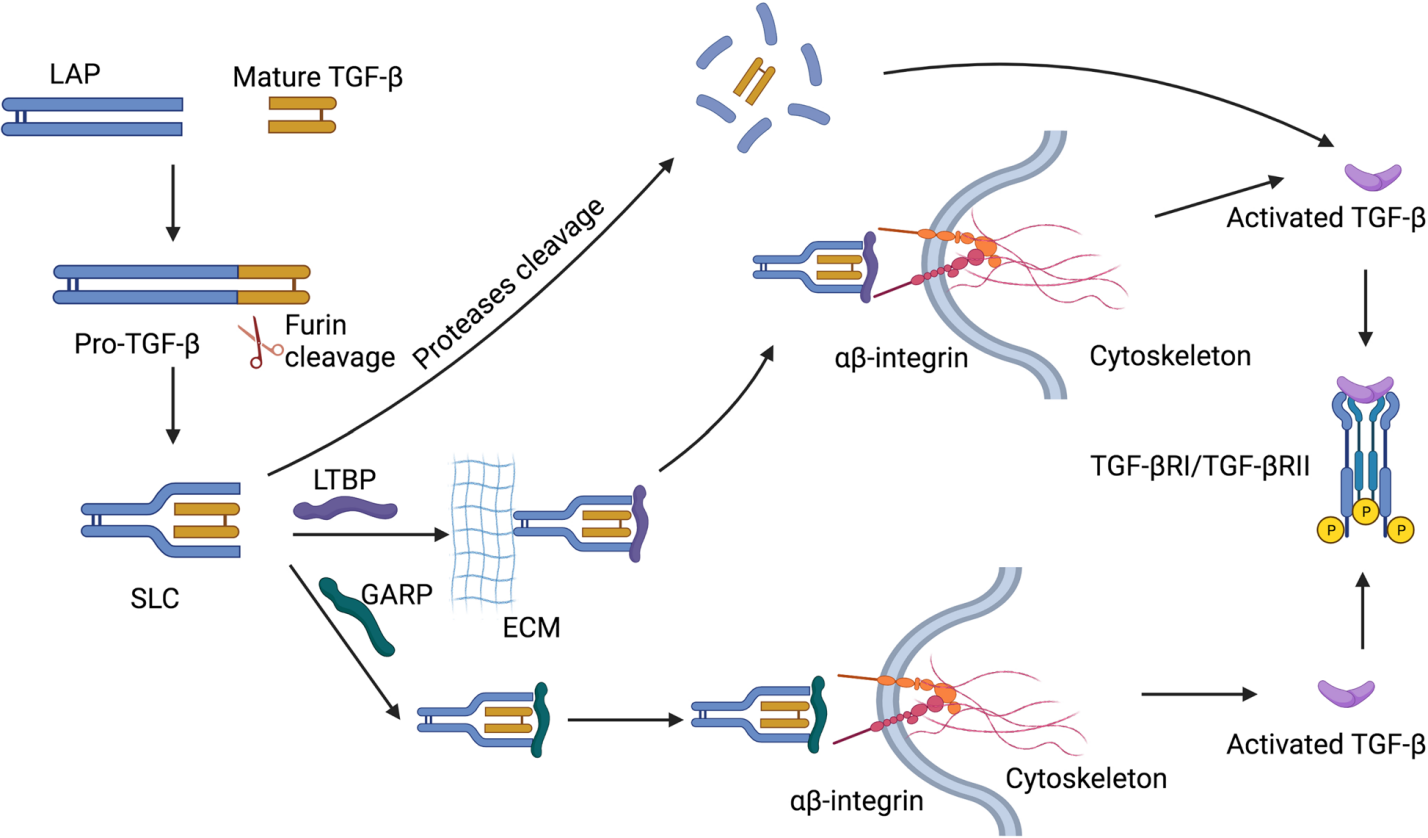
Schematic diagram and activation of latent TGF-β. The pro-TGF-β precursor consists of an N-terminal peptide with latency-associated peptide (LAP) and a mature C-terminal fragment. The pro-TGF-β precursor is cleaved by the convertase furin, and then the LAP dimer binds to mature TGF-β and forms the small latent complex (SLC). Proteases including plasmin, cathepsin and matrix metalloproteinase 9/14 (MMP9/14)) can cleave LAP and release active TGF-β in the extracellular matrix (ECM). SLC binds to latent TGF-β-binding protein (LTBP) with ECM proteins, including fibronectin and fibrillin, mediating the release of active TGF-β via interaction with αβ-integrin. TGF-β can also be activated through SLC anchoring to glycoprotein A repetition predominant protein (GARP)

The mature polypeptides form mature homodimeric and heterodimeric complexes through disulfide-linked dimerization [22]. The latent TGF-β complex is associated with TGF-β binding protein through disulfide bonding to the large latent complex (Fig. 1). The large latent complex is relevant to the extracellular matrix or to the glycoprotein-A repetitions predominant (GARP, also known as leucine-rich repeat containing 32, LRRC32) on the cell surface [23, 24]. Further activation of latent TGF-β is required to release mature TGF-β, which binds to TGF-β receptors on adjacent cells [24]. Besides, TGF-β is supposed to act in a cell- and context-dependent manner [25]. Moreover, latent TGF-β is associated with GARP on mesenchymal stromal cells, platelets, and Tregs, thereby promoting GARP to manage the preservation of these TGF-β complexes [26, 27]. GARP is expressed on fibroblasts, megakaryocytes, and endothelial cells, raising the possibility that GARP plays a broad role in TGF-β1 latency. In addition to GARP, another GARP-related protein, LRRC33, is associated with latent TGF-β and regulates TGF-β activation [23, 28]. Besides, LRRC15, expressed on stromal fibroblasts in advanced tumors, plays similar roles in the cell-associated preservation of TGF-β [29, 30].

### The canonical and noncanonical pathways of TGF-β signal transduction

**Fig. 2:**
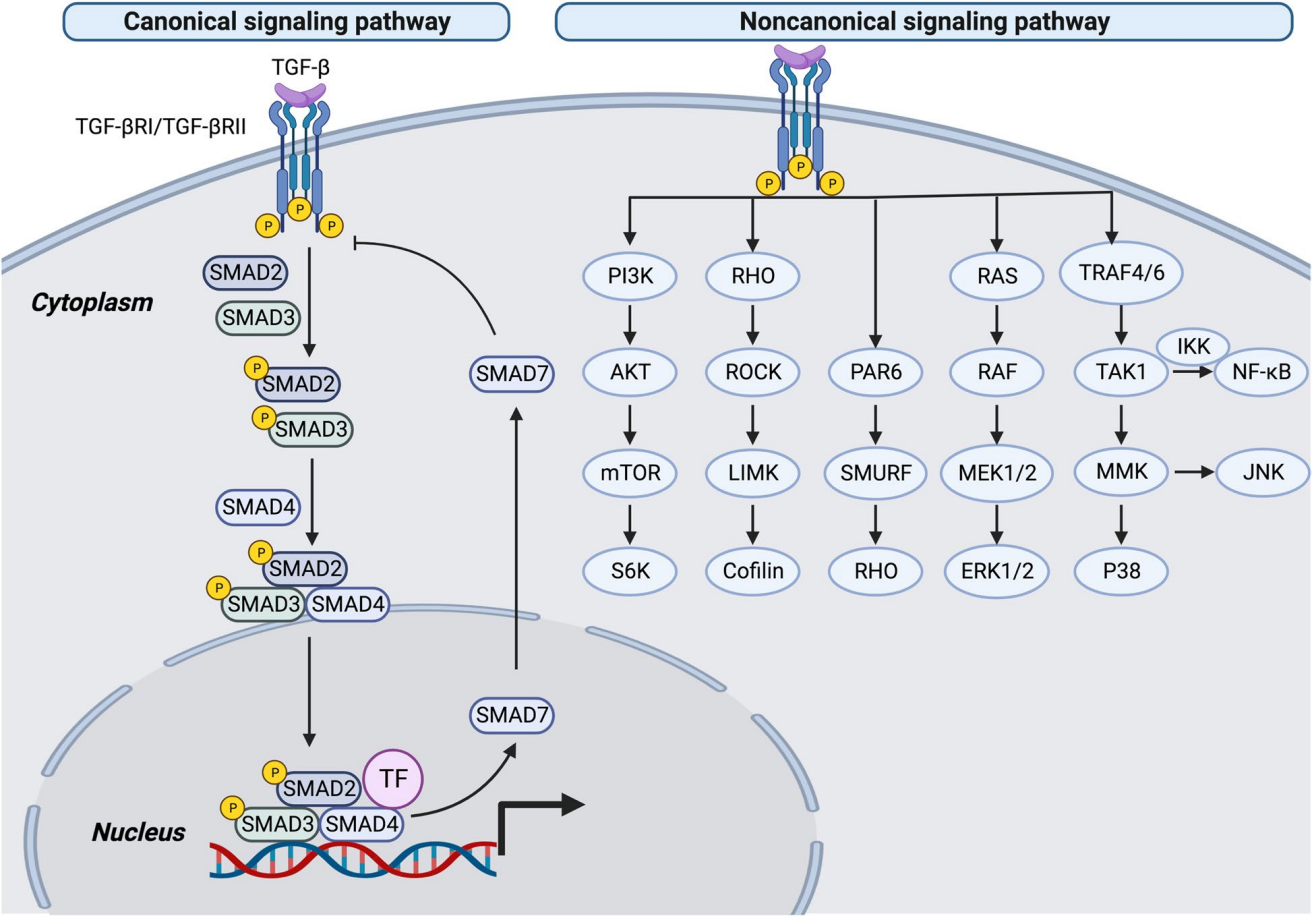
TGF-β induced canonical and noncanonical signaling pathways. TGF-β is presented to TGF-βRII, which phosphorylates TGF-βRI to initiate the subsequent TGF-β pathway. In the canonical pathway, TGF-βRI phosphorylates SMAD2/3 to form the SMAD2/3-SMAD4 complex. SMAD2/3, SMAD4 and transcription factor (TF) complexes are transferred to the cell nucleus, modulating the expression of target genes. SMAD7, one of the target genes, regulates the duration and intensity of TGF-β with a negative feedback loop. In the noncanonical pathway, TGF-β can activate PI3K, RHO, PAR6, RAS, TRAF4/6, JNK, P38, NF-κB and ERK signaling

TGF-β signal transduction depends on canonical and noncanonical pathways (Fig. 2). For the canonical pathway, TGF-β ligands primarily bind to the TGF-β type III receptor (TGF-βRIII, also called betaglycan), which has a high expression level on many cell types. Among the three isoforms of TGF-β, TGF-β2 primarily depends on TGF-βRIII for signaling compared with the other two isoforms [31]. The receptor complex of TGF-β is a tetramer composed of two paired serine or threonine protein kinases, TGF-βRIs and TGF-βRIIs [32]. After binding to TGF-βRIII, TGF-βRIII presents TGF-β to the TGF-βRI/TGF-βRII complex, which has a high affinity for TGF-β [33]. In addition, TGF-β binding to TGF-β can recruit and phosphorylate TGF-βRI, which is a requirement for signal transduction [34]. TGF-βRI phosphorylates SMAD2 on a carboxyl-terminal fragment which contains three serine residues specially at positions 465 and 467 [35]. Then, the phosphorylated SMAD2/3 separates immediately from TGF-βRI and aggregates with SMAD4 to form a heteromeric complex. The formative SMAD2/3-SMAD4 complex translocate into the cell nucleus and activates or restrains target gene expression [36]. TGF-β induced SMAD7 to encode a negative regulator of TGF-β/SMAD signals, which is associated with TGF-βRI, thereby blocking SMAD2 phosphorylation and activation. Moreover, SMAD7 antagonizes TGF-β signals by affecting the formation of the SMAD-DNA complex in the nucleus [37] and inhibits the formation and translocation of the SMAD2-SMAD4 complex [38, 39]. SMAD7 is demonstrated to form complex with SMAD2/3 to mitigate signaling. SMAD7 affects the TGF-β signaling cascades by deactivation of SMAD2/3 and non-SMAD pathways, without any influences on TGF-β receptor activity. SMAD7 is demonstrated to induce myofibroblasts as an endogenous TGF-β-related negative feedback mechanism which inhibits postinfarction fibrosis by restraining TGF-β-independent fibrogenic functions [40]. Overexpression of SMAD7 is associated with inflammatory diseases and is regarded as an inhibitor of TGF-β1 activity [41]. SMAD7 recruits E3 ubiquitin ligases, including tripartite motif-containing protein 31 (TRIM31), SMAD ubiquitination regulatory factor 1/2 (Smurf1/2), and neural precursor cell expressed developmentally downregulated 4–2 (NEDD4–2) to TGF-βRI, promoting proteasomal or lysosomal degradation [42–44]. TGF-βRI ubiquitination is reversed by deubiquitinating enzymes, such as ubiquitin carboxyl-terminal hydrolase L1 (UCHL1) and ubiquitin-specific protease 4 (USP4) [45, 46].

In addition to the canonical signal transduction of TGF-β, the noncanonical signaling pathways play vital roles in diseases [47]. TGF-βRI is demonstrated to activate RHO small GTPases, which control the activity of LIM kinase (LIMK) and phosphorylate cofilin, thereby reorganizing the actin cytoskeleton and participating in cell adhesion and proliferation [48, 49]. TGF-βRII phosphorylates the cell polarity regulator PAR6 and is associated with tight junctions and epithelial-to-mesenchymal transition (EMT) [50, 51]. TGF-β is demonstrated to activate the c-Jun N-terminal kinase (JNK) and P38/mitogen-activated protein kinase (MAPK)/nuclear factor kappa-B (NF-κB) pathways, which are downstream of tumor necrosis factor-associated factor 4/6 (TRAF4/6) [52–54]. PI3K/AKT pathway is also activated as the downstream signal transduction of noncanonical TGF-β signals [55, 56]. In addition, TGF-β induced the phosphorylation of Src homology domain 2-containing protein and then activated the rat sarcoma signal (RAS), rapid accelerated fibrosarcoma signal (RAF), MAPK, and extracellular signal regulated kinase (ERK) pathways [57–59]. RAS-responsive element-binding protein 1 (RREB1) is supposed to provide a connection between RAS and TGF-β signals that coordinate the initiation of fibrogenic EMT [60]. There is crosstalk between the canonical and noncanonical signals induced by TGF-β, which is regulated by receptor tyrosine kinases. TGF-β activated the above pathways by influencing the expression of platelet-derived growth factor (PDGF) in a paracrine or autocrine manner [61].

## The function of TGF-β signals in disease

### TGF-β and the tumor microenvironment (TME)

**Fig. 3:**
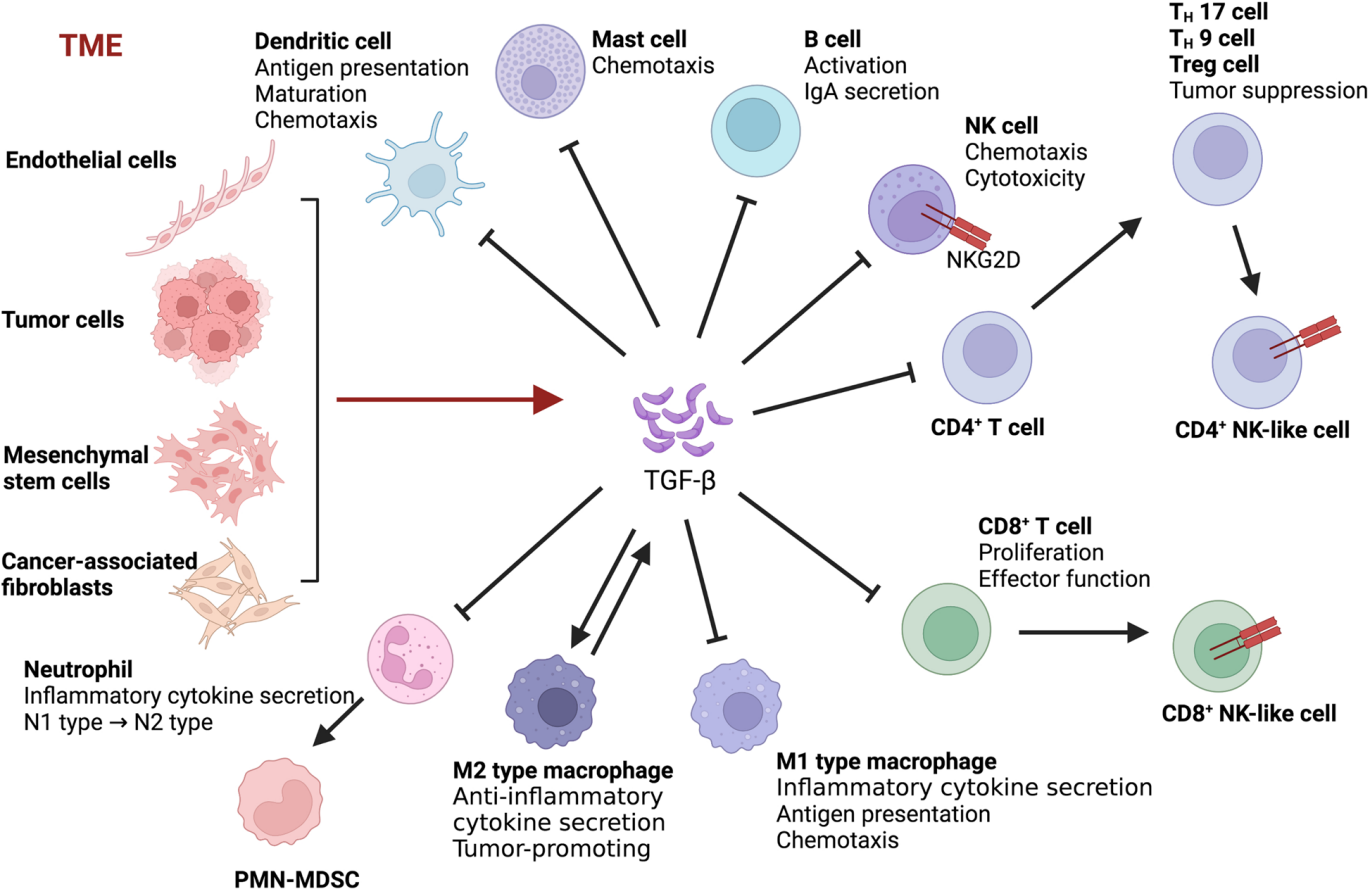
The functions of TGF-β in the TME. Tumor cells, endothelial cells, mesenchymal stem cells, cancer-associated fibroblasts, and macrophages can induce the production and secretion of TGF-β in the TME. TGF-β suppresses tumor immune responses by modulating the multiple functions of immune cells in the TME, as depicted

TGF-β signals play critical roles in the regulation of the TME, which has a complex impact on the progression of cancers (Fig. 3). TGF-β may be used as a biomarker in cancer [62]. The TME contains various types of immune cells, such as tumor-associated macrophages (TAMs), neutrophils, myeloid-derived suppressor cells (MDSCs), dendritic cells (DCs), T cells and B cells, and nonimmune cells, including cancer-associated fibroblasts (CAFs) and stromal cells, as well as a wide range of cytokines [63–66]. Some immunosuppressive cells, such as TAMs and MDSCs, accumulate early in the TME during tumor growth and suppress the T-cell responses that maintain an immunosuppressive environment [67, 68]. In turn, immune cells and stromal cells in the TME are the primary sources of cytokines, including TGF-β [69]. In fact, TGF-β plays a dual role during tumor progression, functioning as a tumor suppressor in the early stage of cancer and as a tumor promoter in the late stages of cancer, such as breast cancer, hepatocellular carcinoma, lung cancer and pancreatic cancer [70–73]. Generally, TGF-β inhibits the proliferation of immunosuppressive myeloid cells, especially in early-stage cancers [74, 75]. In advanced tumors, TGF-β produced by myeloid cells suppresses antitumor immunity and then promotes tumor metastasis [76, 77].

TGF-β inhibited naive CD4^+^ T cells from differentiating into other effector subtypes, such as Tregs, thereby suppressing the antitumor immune response [78]. Depletion of TGF-βRII in CD4^+^ T cells inhibited tumor progression, which resulted in tumor cell death in distant avascular regions due to vascular remodeling [79]. DCs are antigen-presenting cells that deliver tumor antigens to natural killer cells (NK cells) and T cells, inducing antitumor cytotoxic effects [80, 81]. TGF-β blocks cytotoxic CD8^+^ T-cell activation and maturation by inhibiting DC tumor antigen presentation. In addition, TGF-β also inhibited the proliferation and function of CD8^+^ T cells by reducing the secretion of interferon-γ (IFN-γ) and interleukin-2 (IL-2) [82, 83]. TGF-β promoted the expression of antigen-induced programmed death 1 (PD-1) on CD8^+^ T cells, leading to the exhaustion of T cells [84]. TGF-β signals maintain the immunosuppressive properties of CD8^+^ Treg cells. TGF-β and the transcription factor eomesodermin, which controls the follicular location of CD8^+^ Tregs, synergistically promote homeostasis in CD8^+^ Tregs [85]. In addition to influencing T cells, TGF-β regulates the activation, proliferation, and apoptosis of B cells. However, this effect of TGF-β on the B-cell-mediated antitumor immune response has not been well studied [86, 87].

Angiogenesis is a hallmark of cancer during its growth and distant metastasis. Besides, by suppressing the immune system, TGF-β also induces tumor angiogenesis. Increasing evidence shows that tumor angiogenesis is regulated by various cytokines, including TGF-β, IL-22, IL-1β [88–91]. Increased TGF-β expression in the TME is associated with tumor neovascularization in cancers [17, 92]. In endothelial cells, increasing TGF-β1/SMAD3-associated thrombospondin-4 mediated the effects of TGF-β1 on angiogenesis, resulting in tumor growth [93]. An in vivo study showed that increased TGF-β plasma concentrations are related to tumor vascularity [94]. During the tumor process, TGF-β stimulates angiogenesis by affecting TGF-β sequestration [95, 96]. In more detail, fibrillins play an essential role in matrix sequestering of TGF-β. The activation of TGF-β is closely related to integrin binding, which is upregulated upon TGF-β exposure. Moreover, the exposure of fibrillin-2 in the tumor endothelium directly induces tumor angiogenesis by affecting TGF-β sequestering by microfibrils, which results in the higher TGF-β concentration in the TME. Non-polymerized fibrillin-1, fibrillin-2, and fibrillin-containing microfibrils can indirectly bind and sequester TGF-β by interacting with LTBPs, which is the important component of TGF-β and promotes the binding to microfibrils in the ECM [97].

### TGF-β and fibrosis

**Fig. 4:**
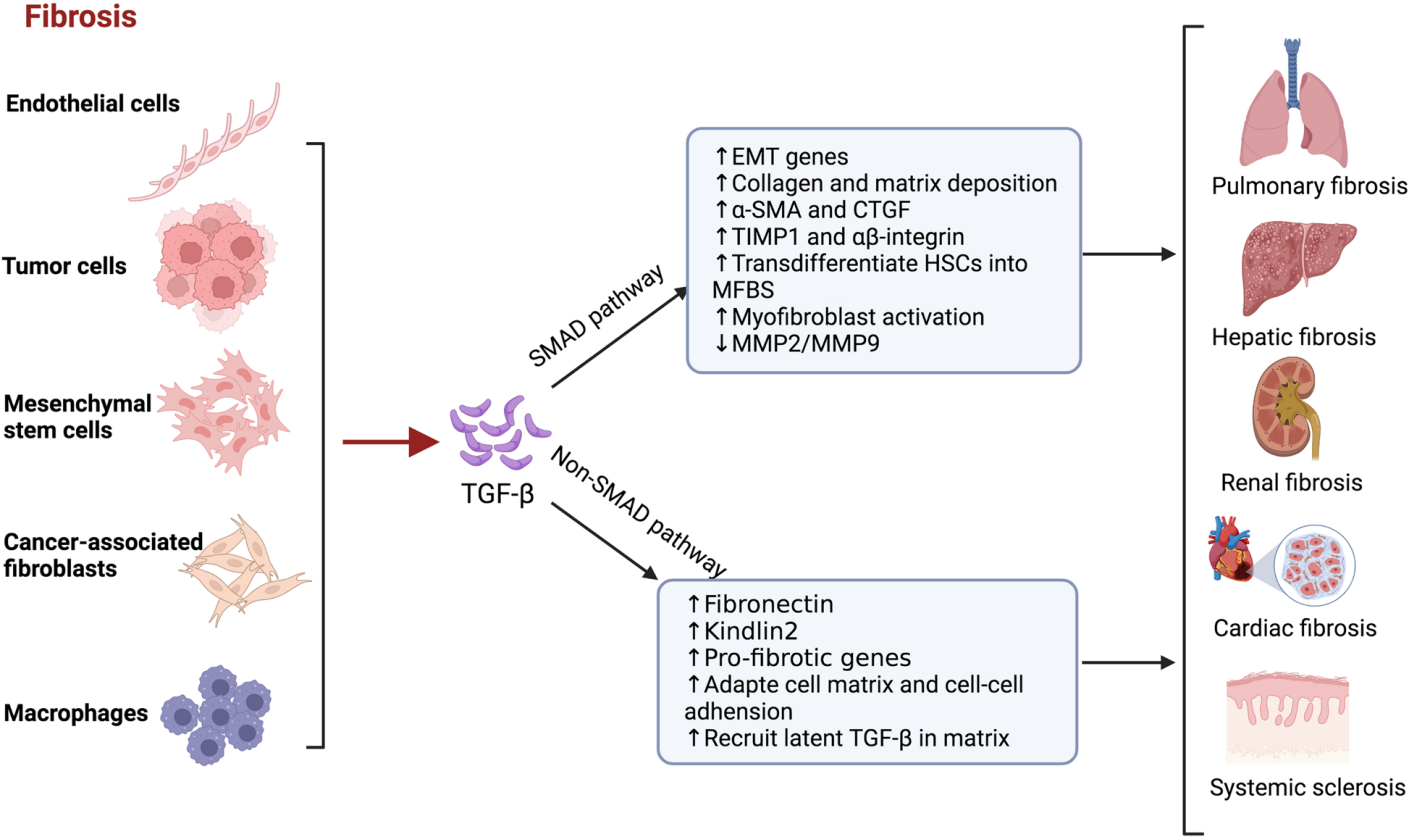
The functions of TGF-β in fibrosis. Tumor cells, endothelial cells, mesenchymal stem cells, cancer-associated fibroblasts, and macrophages can induce the production and secretion of TGF-β, which induces fibrosis, including pulmonary fibrosis, hepatic fibrosis, renal fibrosis, cardiac fibrosis and systematic sclerosis, through SMAD and non-SMAD pathways

Overexpression of TGF-β correlated with the formation and development of fibrosis, which supports the fact that TGF-β is related to fibrotic diseases, such as pulmonary fibrosis, hepatic fibrosis, renal fibrosis, cardiac fibrosis, and systemic sclerosis [7, 98, 99] (Fig. 4). Macrophages are innate immune cells that have essential roles in tissue repair. TGF-β signaling is relevant to resident immune cells, including macrophages, which play critical roles and contribute to the development of fibrosis [100, 101]. TGF-β is crucial in regulating the recruitment and function of macrophages in fibrotic lesions. It functions as a chemoattractant for macrophages, leading to the recruitment of macrophages to fibrotic lesions [102, 103]. In turn, TGF-β induces the secretion of profibrotic cytokines by macrophages, thereby boosting TGF-β activities [104]. Besides. TGF-β also stimulates the expression of ECM proteins by macrophages [105, 106].

ECM deposition is the main characteristic and initial process during fibrosis. TGF-β stimulates the activation and proliferation of fibroblasts, leading to ECM deposition and abnormal organ functions. During this physiological and pathological process, fibroblasts are the main cell types. TGF-β influences the biological behavior of fibroblasts, and low levels of TGF-β promote their proliferation [107]. In addition, TGF-β is chemoattractant for fibroblasts even at a relatively low concentration, which results in recruitment of fibroblasts to fibrotic sites after the activation of TGF-β [108]. Mammalian target of rapamycin (mTOR) signals in the noncanonical pathway are essential in enhancing protein synthesis and activating fibroblasts [109, 110]. Moreover, TGF-β induces epithelial-mesenchymal transition, which contributes to fibroblasts in fibrotic disease [111, 112]. Mechanistically, administration or expression of TGF-β induced fibrosis [113, 114], while inhibiting TGF-β receptor or SMAD signaling decreased the development of fibrosis [115, 116]. SMAD signals cooperated with other signals and transcription factors to promote fibrosis [117], as TGF-β/SMAD signals control the transcription of high-affinity DNA-binding factors, such as TCF/LEF and β-catenin. TCF/LEF and β-catenin are activated by WNT signals and adaptor protein 1 complexes. The adaptor protein 1 complexes are activated by the ERK, JNK, and MAPK pathways [4, 118, 119].

The increase in TGF-β activated fibroblasts, leading to the enhancement of protein synthesis and altering metabolic gene expression [120]. Genes that encode fibronectin and collagen Iα1 are potential transcriptional targets of TGF-β [121, 122]. Moreover, TGF-β promotes the expression of regulators and glycolytic enzymes of metabolism, which results in hyperglycolysis. Meanwhile, the expression of the transcription factor ATF4 increases protein synthesis to meet the crucial needs of collagen and ECM protein synthesis depending on SMAD and mTOR signals [123, 124].

The downstream target genes of TGF-β contributing to the formation of fibrosis is prominent. The crosstalk between the tyrosine kinase receptors and TGF-β signals induced a contractile protein expression signature. This signature leads to α-smooth muscle actin expression, which activates myofibroblast differentiation [125–127]. TGF-β signals are also associated with the expression of connective tissue growth factor (CTGF/CCN2), which plays an essential role in the expression of ECM proteins and the differentiation of myofibroblasts [128, 129]. TGF-β induces the expression of interleukin-11, which is a profibrotic cytokine secreted by fibroblasts and epithelial cells and contributes to myofibroblast differentiation, fibroblast activation, and ECM deposition [130, 131]. TGF-β signal also increases the expression of c-JUN, JUN-B, and JUN-D transcription factors, which heterodimerize with c-FOS and related proteins to form AP-1 transcription complexes, positioning them as drivers of fibrosis. AP-1 complexes are activated in response to ERK, JNK, and MAPK signals induced by TGF-β, thereby promoting fibrosis [132–135]. In addition, TGF-β/SMAD complexes cooperate with AP-1 complexes to increase target gene expression, including those encoding c-JUN, interleukin-11, fibronectin, and collagen Iα2, contributing to fibrosis [136, 137].

TGF-β differentiates cultured tubular epithelial cells into upregulated collagen cells and exhibits a distinct myofibroblast morphology [138–140]. Both canonical (SMAD3-dependent) and noncanonical signals mediate these differentiations [141–144]. TGF-β interacts with β-catenin, which regulates EMT via cAMP response element-binding protein [145]. In addition, bone morphogenic protein-7 (BMP-7) prevents TGF-β-induced EMT in epithelial cells by antagonizing TGF-β, inducing upregulation of α-SMA and downregulation of E-cadherin [139, 146]. TGF-β activates Jagged 1/Notch signals via SMAD and ERK pathways to initiate EMT [147]. TGF-β induces vascular endothelial cells to have mesenchymal characteristics [148–150]. Increased TGF-β signals promote endothelial-mesenchymal transdifferentiation, similar to EMT [151, 152]. Overexpression of TGF-β induces αvβ6 integrin-mediated activation of latent TGF-β in epithelial cells, which plays an essential role in the formation and development of fibrosis via mesenchymal traits [153–155]. Single-cell sequencing of pulmonary fibrotic lesions reveals that cells have suppressive epithelial features and potential mesenchymal characteristics, suggesting the contributions of EMT and endothelial-mesenchymal transdifferentiation to fibrosis [156, 157].

### TGF-β and anemia

The TGF-β superfamily is associated with multiple ineffective erythropoiesis-induced anemias, including myelodysplastic syndrome, Fanconi anemia, β-thalassemia, cancer cachexia-related anemia, acquired aplastic anemia and sickle cell anemia [158–163]. In the hematopoietic system, the TGF-β pathway controls diversified biological processes, ranging from immune system homeostasis to hematopoietic stem cell proliferation, differentiation and self-renewal [164, 165]. The TGF-β/SMAD pathway plays an essential role in ineffective erythropoiesis, which is characterized by early-stage erythroid precursor expansion and late-stage precursor apoptosis [166, 167]. Various cells in the bone marrow niche produce TGF-β, including Schwann cells and megakaryocytes, to maintain the quiescence of hematopoietic stem cells [168, 169]. In addition, transcriptional intermediary factor 1γ (TIF1gamma) induces a differentiation response in hematopoietic stem cells, and SMAD4 mediates the antiproliferative response, whereas SMAD2/3 participates in both of these responses. Overall, SMAD2/3-SMAD4 and SMAD2/3-TIF1gamma are complementary effector arms in controlling hematopoietic cell fate through TGF-β signals [170, 171].

Myelodysplastic syndrome is a hematopoietic stem cell disease that manifests as bone marrow dysplasia and cytopenias because of impaired hematopoiesis [172, 173]. In myelodysplastic syndrome, TGF-β signaling controls the behavior of hematopoietic stem cells in the bone marrow niche. Moreover, the activation of TGF-β impairs the competitive advantage of normal hematopoietic stem cells, which actually contributes to the selection of early-stage myelodysplastic syndrome-genic clones [174, 175].

β-thalassaemia is a β-globin gene mutation that causes genetic disease, which is characterized by iron-loading anemia and ineffective erythropoiesis [176]. TGF-β is a negative regulatory factor in erythrocyte differentiation and maturation, similar to erythropoietin [177]. Hence, TGF-β is a possible target of β-thalassaemia and has been evaluated in clinical studies [178].

In addition to the aforementioned syndrome-inducing anemia, Fanconi anemia is a genetic DNA repair disorder that is characterized by progressive bone marrow failure and predisposition to malignancy [179]. TGF-β signal-mediated growth inhibition is one of the causes of bone marrow failure in Fanconi anemia by impairing the function of hematopoietic stem and progenitor cells [180, 181]. Hence, TGF-β is a potential target of Fanconi anemia.

### TGF-β signaling and inflammatory diseases

TGF-β is supposed to act as a pro- or anti-inflammatory factor contributing to host defense which controls physiologic inflammation and immune response [182]. Overexpression and/or activation of TGF-β are observed in persistent inflammation. On the other hand, systemic routing of TGF-β can also prevent inflammatory pathogenesis through multiple mechanisms [183]. TGF-β maintains T cell tolerance to self and innocuous environmental antigens by influencing the differentiation and homeostasis of effector T cells and Tregs. The activity of TGF-β controls inflammatory response balance by targeting pathogens without evoking over immunopathology to healthy tissues [184].

TGF-β is essential in the development and progression of chronic respiratory diseases which is overexpressed in chronic inflammation, fibrosis and viral infection associated respiratory abnormities including asthma, chronic obstructive pulmonary disease and pulmonary fibrosis [185]. Moreover, TGF-β and SMAD4 mediated uncoupling protein-2 downregulation leads to Aspergillus protease associated inflammation in primary bronchial epithelial cells [186]. Besides, TGF-β is involved in the fluid homeostasis and fibrosis in the lung of COVID-19 patients, which may contribute to a potential immunotherapy strategy [187].

Dysregulated TGF-β signal is also observed in patients with inflammatory bowel disease, which is chronic intestinal inflammation, including ulcerative colitis and Crohn’s disease. The dysfunction of TGF-β signal transduction occurs in T-cells and dendritic cells, which leads to spontaneous colitis in vivo. Moreover, the immune homeostasis of host modulated by intestinal microbes depends on TGF-β production [188]. SMAD4 can restrain naive CD8^+^ T cells from becoming pathogenic for the gut to prevent inflammatory bowel disease in a TGF-β-independent manner [189]. However, the over expression of SMAD7 in inflammatory cells makes them unresponsive to TGF-β1 and negatively regulates gut inflammation [190]. Besides, TGF-β knockout mice present a phenotype with severe multiorgan inflammation [191].

TGF-β is also important in protecting keratinocytes from oxidative stress and involves in the wound healing process [192, 193]. The inhibition of TGF-β is demonstrated to accelerate wound closure and reduce scarring [194, 195]. Exogenous SMAD7 below an oncogenic level can mitigate wound healing and skin inflammation defects related to over activation of TGF-β and NF-κB [196].

### TGF-β signaling and other diseases

In addition to the roles of TGF-β signals in cancers, fibrosis, anemia and inflammatory diseases, this signal is associated with the progression of other diseases. TGF-β family plays an essential role in the maintenance of normal blood vessel wall structure [197]. Mutations in TGF-β family components are associated with specific cardiovascular syndromes, such as primary pulmonary hypertension, and hereditary hemorrhagic telangiectasia [198, 199]. TGF-β family mutation associated specific hereditary vascular syndromes include Osler-Rendu-Weber disease, hereditary hemorrhagic telangiectasia, Loeys-Dietz syndrome, Shprintzen-Goldberg syndrome, and Marfan syndrome [200, 201].

Single-cell RNA sequencing reveals that TGF-β signal overexpression is the upstream driver of smooth muscle cells modulation which plays a pivotal role in promoting extracellular matrix substrate modulation and aortic aneurysm progression in Marfan syndrome [202, 203]. The SMAD signaling of TGF-β is essential in maintaining smooth muscle cell phenotype, while the noncanonical signaling pathway like ERK negatively regulates smooth muscle cell phenotype [204]. Moreover, dysregulated TGF-β signal transduction is related to nonhereditary disorders, including atherosclerosis and cardiac fibrosis, by influencing endothelial cells and smooth muscle cells proliferation, differentiation and migration [205].

The epigenetic alterations of TGF-β canonical and non-canonical pathways are related to thoracic ascending aorta dilatation and aortic aneurysm through remodeling of the vascular wall in Loeys-Dietz and Marfan’s syndromes [206, 207]. Aortic valve disease is characterized by elastic fiber fragmentation, fibrosis, and aberrant angiogenesis. Noncanonical TGF-β signals progressively increase over the progression of aortic valve disease, suggesting that TGF-β signals are possible targets in this disease [208]. In a cohort study, platelet expressed TGF-β1 plays a pivotal role in acute coronary syndromes and indicates a prognostic impact of TGF-β1 on clinical outcomes in patients with coronary artery disease [209].

Besides, TGF-β family members play crucial roles in the development and homeostasis of connective tissue and skeletal system [210]. TGFBR1 or TGFBR2 mutations cause increased expression of TGF-β signaling, connective tissue growth factor and phosphorylation of SMAD2, which lead to a syndrome of altered cardiovascular, neurocognitive, craniofacial and skeletal development [211].

## Therapies based on TGF-β signal transduction in disease

**Fig. 5:**
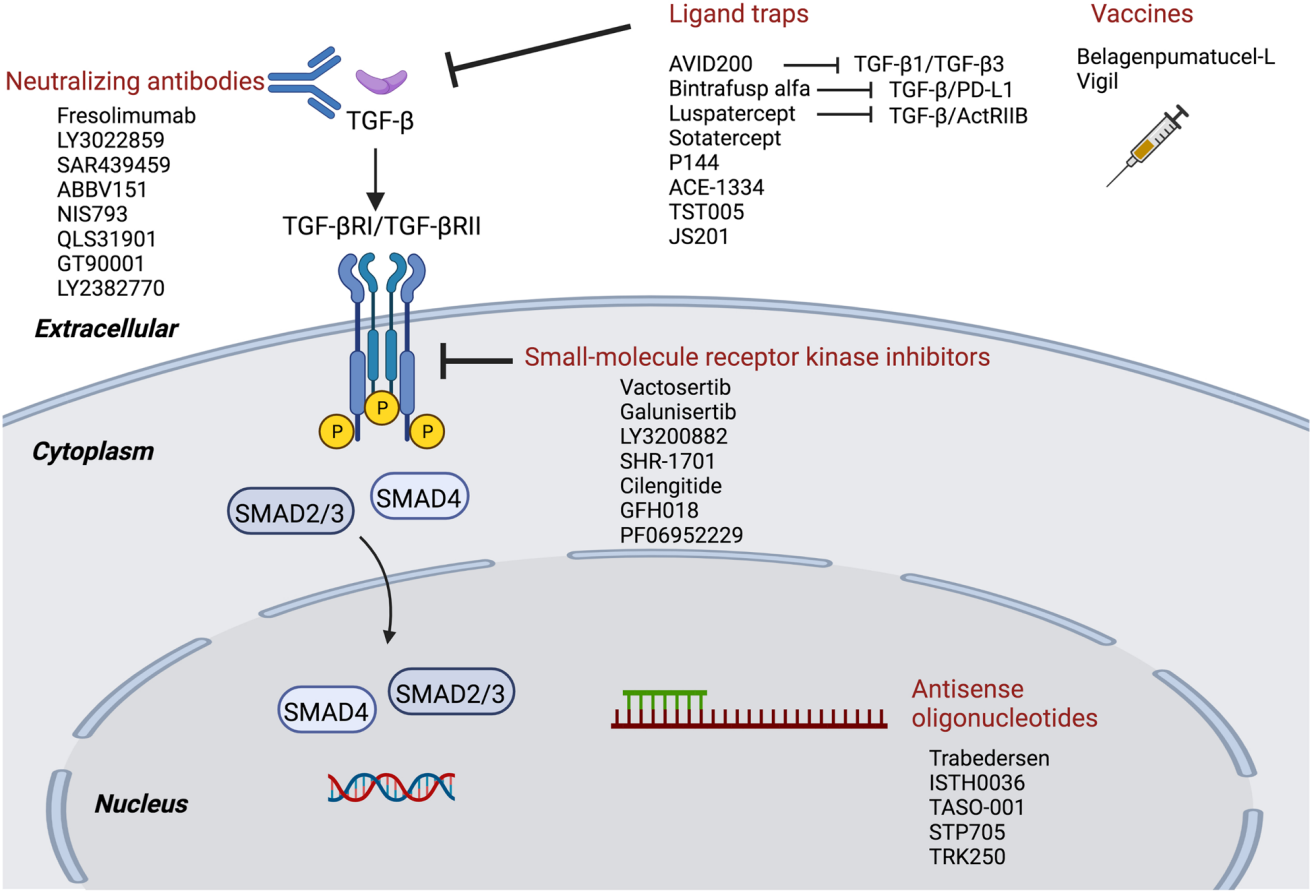
Potential therapeutic strategies based on the TGF-β signaling pathway in disease. Antagonists targeting the TGF-β pathway, including neutralizing antibodies, ligand traps, small-molecule receptor kinase inhibitors, antisense oligonucleotides and vaccines, have recently been evaluated in clinical trials. Representative drugs are shown

Novel strategies targeting TGF-β signaling transduction have been designed and evaluated clinically to treat cancers, sclerosis, and fibrosis. These strategies include neutralizing antibodies and ligand traps, small-molecule receptor kinase inhibitors targeting ligand–receptor signaling pathways, and antisense oligonucleotides to disrupt the production of TGF-β at the transcriptional level. In addition, some vaccines containing a TGF-β antisense transgene, downregulating TGF-β, also show promising therapeutic efficacy in cancer (Fig. 5).

### Neutralizing antibodies

#### Fresolimumab

Fresolimumab (GC1008) is a human IgG4κ anti-TGF-β monoclonal antibody that neutralizes all TGF-β isoforms. This agent is safe and effective in a phase I study for advanced malignant melanoma and renal cell carcinoma. For efficacy, one melanoma patient achieves a partial response, and six patients are proven to have stable disease (NCT00356460) [13]. Fresolimumab potentially controls cutaneous lesions, such as cutaneous keratoacanthomas or squamous cell carcinomas [212]. In addition, administration of fresolimumab during radiotherapy is feasible for patients with metastatic breast cancer. Patients receiving 10 mg/kg fresolimumab have a longer median overall survival with a favorable systemic immune response than patients in the 1 mg/kg group (NCT01401062) [213].

In systemic sclerosis patients, administration of fresolimumab decreases disease-related biomarkers, including THBS1, COMP, SERPINE1, CTGF and other longitudinal pharmacodynamic biomarkers. Regarding efficacy, fresolimumab treatment improves clinical symptoms and decreases the infiltration of dermal myofibroblasts (NCT01284322) [214, 215]. In a clinical trial involving patients with primary focal segmental glomerulosclerosis, fresolimumab is well tolerated [216]. However, an additional phase II study is underpowered and does not achieve the primary or secondary endpoints. Thus, fresolimumab is appropriate for more evaluation in larger studies (NCT01665391) [217]. For osteogenesis imperfecta, a phase I study of fresolimumab is conducted in 8 patients. In this clinical trial, fresolimumab is associated with increases in lumbar spine areal bone mineral density in participants (NCT03064074) [218].

#### LY3022859

LY3022859 is a human anti-anti-TβRII IgG1 monoclonal antibody that inhibits the activation of receptor-mediated signals and has favorable antitumor efficacy for primary tumors and metastatic disease in tumor models [219]. A phase I study including patients with advanced solid tumors shows that the maximum tolerated dose of LY3022859 is not determined. During dose escalation, when the dose of LY3022859 is greater than 25 mg, patients have worsening symptoms, partially due to uncontrolled cytokine release (NCT01646203) [220].

#### *SAR439459*

SAR439459, a neutralizing antibody targeting all isoforms of TGF-β, is supposed to block TGF-β/SMAD signals. This agent also shows activity in reversing TGF-β-mediated NK-cell and T-cell suppression. An in vitro study shows that SAR439459 synergizes with an anti-PD1 antibody, resulting in enhancement of the T-cell response. Moreover, administration of SAR439459 prevents tumor growth by augmenting the proliferation of intertumoral CD8^+^ T cells, reducing their exhaustion, and evoking proinflammatory cytokines in syngeneic tumor models. This evidence supports the ongoing clinical exploration of SAR439459 in patients with solid tumors (NCT03192345) [221].

Other anti-TGF-β neutralizing antibodies, including ABBV151, NIS793, QLS31901, GT90001, and LY2382770, are undergoing clinical trials (Table 1).

**Table 1:** Clinical trials that evaluated TGF-β signaling pathways antibodies and ligand traps in disease

Agent	Targets	ClinicalTrials.gov Identifier	Indication	Number of Patients	Phase	Treatment	Status
***Antibodies***							
ABBV151	GARP/TGF-β1	NCT03821935	Solid tumor	260	Phase I	Monotherapy/+Budigalimab	Recruiting
SAR439459	TGF-β1, β2, β3	NCT05231668	Osteogenesis imperfecta	24	Phase I	Monotherapy	Recruiting
NIS793	TGF-β IgG2	NCT04935359	Pancreatic cancer	501	Phase III	+Chemotherapy	Recruiting
		NCT04390763	Pancreatic cancer	161	Phase II	+Chemotherapy	Recruiting
		NCT04952753	Colorectal cancer	266	Phase II	+Standard of care	Recruiting
		NCT05417386	Pancreatic cancer	50	Phase I	+Chemotherapy	Recruiting
		NCT04810611	MDS	90	Phase I	Monotherapy/+MBG453	Recruiting
QLS31901	PD-L1/TGF-β	NCT04954456	Solid tumor	96	Phase I	Monotherapy	Recruiting
GT90001	ALK-1	NCT03893695	HCC	20	Phase I/II	+Nivolumab	Active, not recruiting
		NCT04984668	Solid tumor	216	Phase I/II	+PD-L1-CTLA-4 bispecific antibody KN046	Recruiting
		NCT05178043	HCC	105	Phase II	+Nivolumab	Recruiting
***Ligand traps***							
Bintrafusp alfa (M7824)	PD-L1/TGF-β RII	NCT04396886	Nasopharyngeal carcinoma	38	Phase II	Monotherapy	Active, not recruiting
		NCT05005429	Pleural mesothelioma	47	Phase II	Monotherapy	Recruiting
		NCT04349280	Urothelial cancer	25	Phase I	Monotherapy	Active, not recruiting
		NCT04396535	NSCLC	80	Phase II	+Docetaxel	Active, not recruiting
		NCT05145569	Ovarian cancer	33	Phase I	+Carboplatin/paclitaxel	Not yet recruiting
		NCT05061823	Lung cancer	42	Phase III	Monotherapy	Recruiting
		NCT04874311	Soft-tissue sarcoma	80	Phase II	+Doxorubicin	Recruiting
		NCT04246489	Cervical cancer	146	Phase II	Monotherapy	Active, not recruiting
		NCT04878250	Bladder cancer	49	Phase II	Monotherapy	Not yet recruiting
		NCT05445882	Prostate cancer	28	Phase II	+N-803 + BN-Brachyury	Not yet recruiting
		NCT05012098	Olfactory neuroblastoma	32	Phase II	Monotherapy	Recruiting
		NCT04708470	Advanced cancer	80	Phase II	+Entinostat and M9241	Recruiting
		NCT04789668	Intracranial metastases	36	Phase I/II	+Pimasertib	Recruiting
		NCT04708067	Intrahepatic cholangiocarcinoma	15	Phase I	+Hypofractionated radiation	Recruiting
		NCT04417660	Thymic carcinoma	38	Phase II	Monotherapy	Recruiting
JS201	PD-1/TGF-βRII	NCT04951947	SCLC	30	Phase II	+Lenvatinib	Recruiting
		NCT04956926	Solid tumor	244	Phase I	Monotherapy	Recruiting
AVID200	TGF-β1, β3	NCT03834662	Solid tumor	19	Phase I	Monotherapy	Active, not recruiting
		NCT03831438	Systemic sclerosis	24	Phase I	Monotherapy	Active, not recruiting
TST005	PD-L1/TGF-β	NCT04958434	Solid tumor	55	Phase I	Monotherapy	Recruiting
ACE-1334	TGF-β1/c3	NCT04948554	Systemic sclerosis	210	Phase I/II	Monotherapy	Active, not recruiting
Luspatercept (ACE-536)	TGF-β	NCT04064060	MDS, β-thalassemia, Myelofibrosis	665	Phase III	Monotherapy	Recruiting
		NCT04477850	MDS	30	Phase II	Monotherapy	Recruiting
		NCT04717414	Myelofibrosis	309	Phase III	Monotherapy	Recruiting
		NCT03900715	MDS	21	Phase II	Monotherapy	Active, not recruiting
		NCT03682536	MDS	350	Phase III	Monotherapy/Epoetin alfa	Recruiting
		NCT04143724	β-Thalassemia	54	Phase II	Monotherapy	Recruiting
		NCT05181735	MDS	150	Phase I/II	Monotherapy/+Epoietin alfa	Recruiting
		NCT05181592	MDS	70	Phase III	Monotherapy	Recruiting
		NCT04539236	MDS	50	Phase I/II	+Lenalidomide	Recruiting
		NCT05384691	MDS	213	Phase II	Monotherapy	Not yet recruiting
Sotatercept (ACE-011)	TGF-β	NCT04796337	PAH	700	Phase III	Monotherapy	Recruiting
		NCT04945460	PAH	180	Phase II	Monotherapy	Recruiting
		NCT04896008	PAH	200	Phase III	Monotherapy	Recruiting
		NCT04811092	PAH	662	Phase III	Monotherapy	Recruiting

*MDS* Myelodysplastic syndrome, *HCC* Hepatocellular carcinoma, *NSCLC* Non-small cell lung cancer, *SCLC* Small cell lung cancer, *PAH* Pulmonary arterial hypertension, *PD-1* Programmed death 1, *PD-L1* Programmed death ligand 1, *CTLA-4* Cytotoxic T lymphocyte antigen 4

### TGF-β ligand traps

#### AVID200

TGF-β ligand traps are chimeric fusion proteins designed to restrain TGF-βs from binding to TGF-β receptors based on their ectodomain. AVID200 is a potent TGF-β1/TGF-β3 protein trap that enhances antitumor efficacy in a syngeneic 4 T1 triple-negative breast cancer model [86]. Currently, a phase I clinical trial of AVID200 has been conducted for advanced solid tumors (NCT03834662).

In fibrotic disease, administration of AVID200 decreases the proliferation of human mesenchymal stromal cells and reduced the phosphorylation of SMAD2 and the expression of collagen. Myelofibrosis mononuclear cells present increasing progenitor cells emerging after treatment with AVID200. In addition, AVID200 treatment reduces bone marrow fibrosis, increases bone marrow cellularity, and increases the numbers of murine progenitor and hematopoietic stem cells in a myelofibrosis mouse model [222]. AVID200 is supposed to promote the survival of murine/human fanconi anemia hematopoietic stem and progenitor cells in vitro by downregulating nonhomologous end-joining pathway-related genes and reducing DNA damage in vivo [223]. AVID200 also increases the hematopoietic colony formation of Shwachman-Diamond Syndrome patients’ bone marrow, leading to the improvement of bone marrow failure [224]. Currently, a clinical trial of AVID200 for systemic sclerosis has been launched (NCT03831438).

#### Bintrafusp alfa

Bintrafusp alfa (M7824) is a bifunctional fusion protein that contains the extracellular TGF-β trap fused to a human IgG monoclonal antibody against PD-L1. Bintrafusp alfa synergizes effectively with radiotherapy by modulating the TME to reverse cancer immune evasion. Combining bintrafusp alfa with radiotherapy increases tumor-infiltrating lymphocytes, attenuates radiotherapy-induced fibrosis, reconstitutes tumor immunity and regresses spontaneous lung metastases [12]. In addition, bintrafusp alfa shows safety and clinical activity in human papillomavirus (HPV)-associated cancers. The objective response rate is 30.5%, including five patients, with a disease control rate of 44.1% (NCT02517398, NCT03427411) [225]. Bintrafusp alfa is safe and enhances tumor antigen-specific immunity by reversing Treg immunosuppression and reducing myeloid cell tumor infiltration in patients with HPV-unrelated head and neck squamous cell carcinoma [226]. Several factors are associated with the clinical response during bintrafusp alfa therapy, including low levels of TGF-β1 expression and higher CD8^+^ T cell: MDSC ratios [227]. Bintrafusp alfa has promising antitumor efficacy in a phase I study involving patients with non-small cell lung cancer who are previously treated with platinum. The objective response rate in all patients is 21.3% (NCT02517398) [228]. In a phase I trial, bintrafusp alfa also has clinical activity for biliary tract cancer, with an objective response rate of 20%. In addition, the overall survival is 12.7 months [229]. In patients with advanced esophageal adenocarcinoma and esophageal squamous cell carcinoma, bintrafusp alfa shows clinical antitumor efficacy with a manageable safety profile. In patients with esophageal adenocarcinoma, the confirmed objective response rate is 20.0% (NCT02517398) [230]. Similarly, the confirmed objective response rate is 10.0% in patients with esophageal squamous cell carcinoma, with a median overall survival of 11.9 months (NCT02699515) [231]. Bintrafusp alfa also has antitumor efficacy in patients with pretreated advanced squamous cell carcinoma of the head and neck. The confirmed objective response rate is 13%, with 4 patients having stable disease (NCT02517398) [232]. In patients with advanced gastric and gastroesophageal junction cancer, the objective response rate to bintrafusp alfa is 16%, with a disease control rate of 26% [233]. In patients with advanced solid tumors who received bintrafusp alfa treatment, two of 23 patients have a partial response, for a disease control rate of 35.7% (NCT02699515) [234].

#### Luspatercept

Luspatercept (ACE-536, reblozyl) is an activin receptor type IIB fusion protein–ligand trap targeting TGF-β/SMAD signals. This agent has been used to treat anemia diseases, including beta-thalassemia, myelofibrosis, and myelodysplastic syndromes [235]. TGF-β/SMAD signals promote erythroid maturation by enhancing the differentiation of late-stage erythroblasts, thereby improving anemia [236]. Luspatercept impacts the bone marrow microenvironment, leading to a selective restoration of ineffective hematopoiesis [237]. In patients with transfusion-dependent lower-risk myelodysplastic syndrome, luspatercept shows clinical activity in a phase II (PACE-MDS) trial and a phase III (MEDALIST) trial, leading to US Food and Drug Administration approval in 2020 [238]. In a phase III (MEDALIST) trial, 38% of the patients treated with luspatercept have transfusion independence for 8 weeks and even longer (NCT02631070) [239–241]. In a phase II (PACE-MDS) trial, luspatercept is well tolerated and has clinical efficacy for patients with myelodysplastic syndromes inducing anemia (NCT01749514, NCT02268383) [242, 243]. In patients with myelodysplastic syndromes or myeloproliferative neoplasms who currently have no effective treatments, administration of luspatercept reduces the transfusion burden and improves the modified hematologic response-erythroid levels [244]. Luspatercept therapy has been demonstrated to strengthen the contribution of host immunity to disease biology in myelodysplastic syndromes with ring sideroblasts [245].

In patients with β-thalassemia after luspatercept therapy in a clinical trial, twenty-six of 64 patients achieve over a 20% reduction in red blood cell transfusion burden (NCT01749540 and NCT02268409) [246]. In a phase III (BELIEVE) trial for transfusion-dependent β-thalassemia, the transfusion burden is reduced after the administration of luspatercept (NCT02604433) [247]. Luspatercept is also supposed to be a potential strategy in patients with nontransfusion-dependent β-thalassemia [248]. In this phase II (BEYOND) trial in patients with nontransfusion-dependent β-thalassemia, 77% of patients after luspatercept therapy achieve an increase in hemoglobin concentration (NCT03342404) [249].

#### Sotatercept

Sotatercept (ACE-011), a TGF-β ligand trap, restrains late-stage negative regulators of erythropoiesis and improves ineffective erythropoiesis. For anemia caused by β-thalassemia, a phase II study demonstrated that sotatercept is clinically efficient and well tolerated. In nontransfusion-dependent patients, 18 of 30 (60%) achieve a hemoglobin increases of more than 1.0 g/dL, which is sustained for more than 3 months. In the transfusion-dependent β-thalassemia subgroup, four (100%) patients achieve a more than 20% transfusion-burden reduction (NCT01571635) [250].

For pulmonary arterial hypertension, a phase II (PULSAR, NCT03496207) study shows a reduction in pulmonary vascular resistance after sotatercept treatment [251]. The extension study revealed that 32 of 97 (30.8%) participants suffer serious treatment-related adverse events. Importantly, the placebo-crossed to sotatercept group is demonstrated to have improved both primary and secondary endpoints. The clinical effectiveness is well maintained in the patients with continued sotatercept [252].

For lower-risk myelodysplastic syndromes, especially in patients for whom previous erythropoiesis-stimulating agents failed, sotatercept is well tolerated and clinically effective. Thirty-six of 74 (49%) patients achieve hematological improvement-erythroid. Among them, 29 of 62 (47%) participants with a high transfusion burden achieve hematological improvement-erythroid, whereas seven of 12 (58%) patients with a low transfusion burden achieve hematological improvement-erythroid (NCT01736683) [253].

In patients with chemotherapy-induced anemia in advanced solid tumors, both clinical trials are terminated early because of the slow patient accrual. However, the existing results indicate that sotatercept is potentially effective with an acceptable safety profile when treated with chemotherapy-induced anemia (NCT00931606, NCT01284348) [254].

#### P144

P144 (Disetertide©) is a peptide inhibitor of TGF-β1. This inhibitor decreases the proliferation and invasiveness of glioblastoma cells. P144 increases apoptosis and anoikis by reducing SMAD2 phosphorylation, downregulating SK, and upregulating SMAD7 in vitro. Additionally, P144 impairs tumor growth and increases survival in a glioblastoma mouse model [255]. Besides, treatment with P144 results in a reduction in the mitotic-to-apoptotic ratio and angiogenesis, which are induced by TGF-β1. In addition, P144 abrogates EMT and the phenotypes of cancer stem cells, which decreases liver metastasis in patients with colorectal cancer [256]. P144 reduces tumor growth by reducing the infiltration of macrophages and increasing the intratumor levels of MCP-1 and VEGF [257]. The therapeutic applications of P144 are limited due to a lack of target selection, possible recognition by the immune system, and potential cytotoxicity on healthy cells. Encapsulation of P144 with nanoparticles facilitated its dissolution, improves its functionalization and improves its potential therapeutic applications in liver cancer [258].

P144 also has treatment efficacy in controlling fibrotic disease. Administration of P144 reduces radiation-induced fibrosis in soft tissue sarcoma by retaining the macro- and microscopic morphology of muscle, reducing extracellular matrix fibrosis and reducing SMAD2/3 phosphorylation [259]. P144 decreases renal fibrosis by blocking TGF-β1/SMAD3 signals and modulating the polarization of macrophages, suggesting its possible therapeutic potential in ischemia–reperfusion injury-induced renal fibrosis [260]. P144 decreases laser-induced choroidal neovascularization in a rat model [261]. P144 is also proposed to promote the maturation of scars, with the improvement of the morphology of hypertrophic scars in a mouse model [262]. P144 prevents the formation of an aortic aneurysm but not its progression in a Mafan syndrome mouse model. Hence, reducing the excess of active TGF-β signaling during the early stages of aortic disease progression is essential [263]. Furthermore, P144 inhibits TGF-β-dependent signals in cardiac fibroblasts, preventing myocardial fibrosis in spontaneously hypertensive rats [264]. P144 also inhibits NADPH oxidases and prevents kidney oxidative stress in spontaneously hypertensive rats [265].

Other TGF-β ligand traps, including ACE-1334, TST005, and JS201, are under evaluation in clinical trials (Table 1).

### Small-molecule receptor kinase inhibitors

#### Vactosertib

Currently, some small-molecule receptor kinase inhibitors of TGF-β signals are undergoing clinical trials to treat cancer and fibrosis. Vactosertib (TEW-7197, EW-7197) is a small-molecule kinase inhibitor of TGF-βRI that has promising antitumor and antifibrotic potential [11, 266, 267]. Vactosertib inhibits hepatic, renal, and pulmonary fibrosis by blocking both TGF-β1/SMAD2/3 and reactive oxygen species (ROS) signals [268]. The combination of vactosertib with radiation has a favorable antimetastatic efficacy in breast cancer [269]. Vactosertib prevents ulcerative colitis-associated inflammation and fibrosis, protecting against postsurgical adhesion formation by downregulating proinflammatory and profibrotic genes, inhibiting oxidative stress, decreasing inflammatory cell infiltration, and inhibiting excessive collagen deposition [270–272]. The combination of vactosertib with imatinib mesylate, a tyrosine kinase inhibitor, delays chronic myeloid leukemia relapse and prolongs survival by eliminating leukemia-initiating cells [273]. Vactosertib potently inhibits breast cancer lung metastasis by inhibiting SMAD/TGF-β signals and enhancing the activity of cytotoxic T cells [274]. Clinical trials based on vactosertib are undergoing in melanoma, lung cancer, urothelial carcinoma, gastric cancer, and colorectal cancer (Table 2).

**Table 2:** Clinical trials that evaluated TGF-β signaling pathways small molecule receptor kinase inhibitors, antisense nucleotides and vaccines in disease

Agent	Targets	ClinicalTrials.gov Identifier	Indication	Number of Patients	Phase	Treatment	Status
***Small molecule kinase inhibitors***							
Vactosertib (TEW-7197)	TGF-βRI	NCT05436990	Melanoma	30	Phase II	+Pembrolizumab	Not yet recruiting
		NCT03143985	Multiple myeloma	18	Phase I	+Pomalidomide	Recruiting
		NCT04064190	UC	48	Phase II	+Durvalumab	Not yet recruiting
		NCT04515979	NSCLC	55	Phase II	+Pembrolizumab	Recruiting
		NCT04103645	Myeloproliferative neoplasms	37	Phase II	Monotherapy	Recruiting
		NCT04893252	Gastric cancer	55	Phase II	+Durvalumab	Not yet recruiting
		NCT04258072	Pancreatic cancer	24	Phase I	+Chemotherapy	Recruiting
		NCT03802084	Desmoid tumor	24	Phase I/II	+Imatinib	Recruiting
		NCT03724851	GC/GEJC	67	Phase I/II	+Pembrolizumab	Active, not recruiting
		NCT04656002	Gastric cancer	43	Phase II	+Paclitaxel+Ramucirumab	Not yet recruiting
		NCT03732274	NSCLC	60	Phase I/II	+Durvalumab	Active, not recruiting
		NCT03844750	Colorectal cancer	19	Phase II	+Pembrolizumab+Hepatectomy	Recruiting
		NCT03698825	Gastric cancer	62	Phase I/II	+Paclitaxel	Active, not recruiting
Galunisertib (LY2157299)	TGF-βRI	NCT02672475	TNBC	29	Phase I	+Paclitaxel	Active, not recruiting
		NCT02452008	Prostate cancer	60	Phase II	+Enzalutamide	Recruiting
		NCT03206177	Carcinosarcoma	26	Phase I	+Paclitaxel/Carboplatin	Active, not recruiting
		NCT02688712	Colorectal cancer	50	Phase II	+Neoadjuvant Chemoradiation	Active, not recruiting
LY3200882	TGF-βRI	NCT02937272	Solid tumor	223	Phase I	Monotherapy	Active, not recruiting
GFH018	TGF-βRI	NCT05051241	Solid tumor	60	Phase I	Monotherapy	Recruiting
		NCT04914286	Solid tumor	195	Phase I/II	+Toripalimab	Recruiting
		NCT05386888	NSCLC	65	Phase II	+Toripalimab	Not yet recruiting
SHR-1701	PD-L1/TGF-βRII	NCT05106023	Melanoma	31	Phase II	+Temozolomide	Not yet recruiting
		NCT05020925	NPC	30	Phase I/II	+Famitinib	Not yet recruiting
		NCT04650633	HNSCC	130	Phase II	Monotherapy	Recruiting
		NCT04624217	Pancreatic cancer	56	Phase I/II	+Chemotherapy	Active, not recruiting
		NCT03710265	Solid tumor	206	Phase I	Monotherapy	Recruiting
		NCT04937972	NSCLC	71	Phase II	+Fluazopalil	Recruiting
		NCT04974957	NSCLC	71	Phase II	+BP102	Not yet recruiting
		NCT05300269	Rectal cancer	73	Phase II	+Radiotherapy and Chemotherapy	Recruiting
		NCT04884009	SCLC	106	Phase II	Monotherapy/+Famitinib	Not yet recruiting
		NCT04580498	NSCLC	122	Phase II	Monotherapy/+Chemotherapy	Not yet recruiting
		NCT05177497	NSCLC	19	Phase II	Monotherapy	Not yet recruiting
		NCT05149807	GC/GEJC	896	Phase II	Monotherapy	Enrolling by invitation
		NCT04856774	Solid tumor	113	Phase I/II	+BP102	Recruiting
		NCT04282070	NPC	91	Phase I	Monotherapy/+Chemotherapy	Active, not recruiting
		NCT04679038	Solid tumor	222	Phase I/II	Monotherapy/+Famitinib	Recruiting
		NCT04856787	Colorectal cancer	439	Phase II	+BP102 and XELOX	Recruiting
		NCT04324814	Solid tumor	48	Phase I	Monotherapy	Active, not recruiting
		NCT05179239	Cervical cancer	572	Phase III	+Chemotherapy+BP102	Recruiting
		NCT04699968	NSCLC	168	Phase II	Monotherapy/+Famitinib	Not yet recruiting
		NCT05132413	NSCLC	561	Phase III	+Bevacizumab and Chemotherapy	Not yet recruiting
		NCT04950322	GC/GEJC	920	Phase III	+Chemotherapy	Recruiting
		NCT04407741	Solid tumor and B-cell lymphomas	100	Phase I/II	Monotherapy/+SHR2554	Recruiting
		NCT04560244	NSCLC	15	Phase II	Monotherapy	Not yet recruiting
***Antisense nucleotides***							
STP705	TGF-β1/COX-2	NCT04669808	Basal cell carcinoma	15	Phase II	Monotherapy	Recruiting
		NCT05421013	Bowen’s disease	30	Phase I/II	Monotherapy	Recruiting
		NCT04844840	Keloid recurrence	60	Phase II	Monotherapy	Recruiting
		NCT05196373	Hypertrophic scar	50	Phase I/II	Monotherapy	Not yet recruiting
		NCT04676633	HCC	50	Phase I	Monotherapy	Recruiting
		NCT04844983	Bowen’s disease	100	Phase II	Monotherapy	Recruiting
***Vaccines***							
Vigil (Gemogenovatucel-T)	TGF-β1, β2	NCT03495921	Ewing’s sarcoma	114	Phase III	+Irinotecan and Temozolomide	Active, not recruiting
		NCT03073525	Gynecological cancers	25	Phase II	+Atezolizumab	Active, not recruiting
		NCT01309230	Ovarian cancer	44	Phase II	Monotherapy	Active, not recruiting
		NCT02346747	Ovarian cancer	91	Phase II	Monotherapy	Active, not recruiting

*UC* Urothelial carcinoma, *NSCLC* Non-small cell lung cancer, *GC/GEJC* Gastric cancer/gastroesophageal junction cancer, *TNBC* Triple-negative breast cancer, *NPC* Nasopharyngeal carcinoma, *HNSCC* Head and neck squamous cell carcinoma, *HCC* Hepatocellular carcinoma

#### Galunisertib

Galunisertib (LY2157299) is another small-molecule inhibitor that selectively binds to TGF-βRI, inhibiting kinase activity [275]. Galunisertib exerts antifibrotic effects on dermal fibroblasts by attenuating the expression of fibrotic genes and increasing the expression of antifibrotic genes such as decorin and MMP1 [276]. Galunisertib is a potential prophylactic drug for treating traumatic heterotopic ossification by intercepting TGF-β/SMAD2/3 signals [277]. What is more, galunisertib shows a prominent antifibrotic potential in liver fibrosis by inhibiting phosphorylation of SMAD2, blocking the production and maturation of collagens, and promoting the degradation of collagens [278, 279].

Galunisertib overcomes stemness-derived aggressiveness by reducing the expression of CD44 and THY1 in hepatocellular carcinoma [280]. A phase IB study of galunisertib plus ramucirumab for advanced hepatocellular carcinoma shows that the combination therapy displays favorable pharmacokinetics, with a disease control rate of 12.5% [281]. A pilot study of galunisertib combined with stereotactic body radiotherapy in patients with advanced hepatocellular carcinoma shows good tolerability and is associated with antitumor activity. Two out of 15 patients achieve a partial response [282]. In a phase II study, galunisertib plus sorafenib results in prolonged overall survival [283]. In patients with unresectable pancreatic cancer, galunisertib plus gemcitabine improves overall survival [284]. In another phase IB clinical trial of patients with pancreatic cancer, galunisertib is coadministered with durvalumab, showing tolerable adverse events but limited clinical activity, with progression-free survival of 1.87 months [285].

Galunisertib is supposed to suppress the activation of SMAD2 in neuroblastomas and activate NK cells, restore NK cytotoxic activity, and increase the efficacy of dinutuximab with activated NK cells against neuroblastoma tumors [286]. For recurrent glioblastoma, the combination of galunisertib and lomustine fails to demonstrate improved overall survival compared with the group receiving monotherapy [287]. In a phase II study of galunisertib for myelodysplastic syndromes, 10 out of 41 patients achieve hematologic improvement erythroid response, 18 patients have erythroid response and nine of 28 transfusion-dependent patients achieve hematologic improvement [288]. Other clinical trials targeting solid tumors, including hepatocellular carcinoma, breast cancer, and glioma, are ongoing (Table 2).

#### LY3200882

LY3200882 is an orally selective next-generation potent adenosine triphosphatase competitive TGF-βRI small-molecule inhibitor that has promising antitumor efficacy [289, 290]. Codelivery of LY3200882 and programmed cell death protein ligand 1 (PD-L1) siRNA boosts antitumor immunotherapy by downregulating the expression of ECM, promoting the infiltration of effector T cells, resulting in enhanced tumor antigen presentation and reversing the immunosuppressive microenvironment in triple-negative breast cancer [289]. LY3200882 effectively inhibits liver metastases by increasing the infiltration of CD8^+^ cytotoxic T cells and inhibiting the recruitment of immunosuppressive cells such as MDSCs in colorectal mouse models [290]. A phase I study showed that LY3200882 is well tolerated, with preliminary antitumor activity in advanced cancer. Four patients with grade 4 glioma have partial responses. In patients with advanced pancreatic cancer, 6 out of 12 patients have partial responses, and 3 patients are stable disease. In this trial, the overall disease-control rate of LY3200882 plus gemcitabine and nab-paclitaxel is 75% [291].

#### SHR-1701

SHR-1701 is a bifunctional fusion protein that is a PD-L1 monoclonal antibody fused with the extracellular TGF-βRII domain. This agent has promising antitumor efficacy in advanced cervical cancer [292, 293]. Among 32 patients with cervical cancer, the objective response rate is 15.6%, and the disease control rate is 50.0%. Notably, as assessed by imRECIST, the median PFS is 4.1 months, and the 12-month overall survival rate is 54.6% (NCT03774979) [293]. Moreover, patients with lung cancer suffering from persistent lymphopenia after chemotherapy are sensitive to SHR-1701 [294].

#### Cilengitide

αvβ integrin is a major local activator of latent TGF-β. Genetically and pharmacologically targeting αvβ integrin inhibits the TGF-β signals and suppresses tumor metastasis [295–297]. Cilengitide, a selective cyclic RGD pentapeptide antagonist of αvβ3 and αvβ5 integrin, has been demonstrated to modulate the attachment and viability of glioma cells and induce autophagy-mediated cell death [298, 299]. In a phase III study of cilengitide plus standard treatment for patients with glioblastoma, combinational therapy does not improve the outcomes [300]. In this trial, the authors recommend a different continuous infusion schedule that is more appropriate according to the pharmacokinetics [301]. In a phase II study with two cilengitide regimens plus standard treatment for patients with glioblastoma, the median overall survival is 16.3 months in the cilengitide arm and 14.5 months in the intensive cilengitide arm (NCT00813943) [302]. However, in another phase II trial, cilengitide plus metronomic temozolomide, procarbazine, and standard radiotherapy does not improve survival in patients with glioblastoma [303].

Cilengitide is supposed to enhance the inhibition of erlotinib on TGF-β1-induced EMT and phosphorylation of SMAD2/3 [304, 305]. In a phase I study, continuous infusion of cilengitide plus chemoradiotherapy for patients with stage III non-small cell lung cancer is potentially tolerable. Four out of 9 patients have a complete response, and 4 patients have a partial response [306]. In another phase II study that combined cilengitide with cetuximab and platinum-based chemotherapy as first-line treatment in advanced non-small cell lung cancer patients, the progression-free survival is 6.8 months in the cilengitide group versus 5.6 months in the control group. The median overall survival is 13.6 versus 9.7 months compared with the control group (NCT00842712) [307].

Cilengitide has been demonstrated to downmodulate the invasiveness of melanoma cells by targeting αvβ5 integrin [308]. Cilengitide is well tolerated but has limited antitumor efficacy as a monotherapy for metastatic melanoma [309, 310]. Cilengitide enhances the effectiveness of anti-PD1 treatment and produces a more robust antitumor immune response by decreasing STAT3 phosphorylation and reducing tumor PD-L1 expression in a melanoma mouse model [311]. In addition, activation of POSTN releases TGF-β1 from the ECM and initiates the POSTN/TGF-β1 positive feedback loop. Cilengitide plus lenvatinib suppresses tumor cell growth in a hepatocellular carcinoma mouse model [312]. Generally, cilengitide, combined with paclitaxel, is well tolerated and has antitumor activity in patients with advanced solid tumors [313].

Cilengitide treatment decreases adhesion to vitronectin and fibronectin and reduces the expression of TGF-β-induced fibronectin genes, as well as the accumulation of mRNAs for fibronectin and collagen type I. However, cilengitide does not inhibit the development of pulmonary fibrosis in vivo [314]. Pharmacological inhibition of integrin utilizing cilengitide in vivo decreases angiogenesis but worsens biliary and septal fibrosis, despite its antifibrogenic effect on hepatic stellate cells [315, 316].

Other small-molecule inhibitors targeting TGF-β signaling pathways, such as GFH018 and PF06952229, are under clinical evaluation for patients with lung cancer, breast cancer, and prostate cancer (Table 2).

### Antisense oligonucleotides (ASOs)

#### Trabedersen

Trabedersen (AP12009) is a synthetic phosphorothioate antisense oligodeoxynucleotide blocking the production of TGF-β2. Trabedersen has therapeutic potential in malignant brain tumors, skin tumors, pancreatic cancer, and colorectal cancer [317]. Trabedersen reduces the secretion of TGF-β2, inhibits cell proliferation and migration, and reverses TGF-β2-mediated immunosuppression in pancreatic cancer. In addition, trabedersen significantly inhibits tumor growth and lymph node metastasis in pancreatic cancer [318]. In a phase II clinical trial for recurrent high-grade glioma, superior efficacy is observed for trabedersen over chemotherapy. This positive risk-benefit assessment demonstrates its clinical use in high-grade glioma [319].

#### ISTH0036

ISTH0036 is an antisense oligonucleotide selectively targeting TGF-β2 signals [320]. In a phase I study involving patients with primary open angle glaucoma who receive trabeculectomy, single-dose ISTH0036 administration at the time of trabeculectomy results in intraocular pressure values persistently less than 10 mmHg during the three-month postoperative period [15]. Future phase II clinical trials are needed to assess repeat dosing for up to one year for constant antifibrotic effects. It is critical for clinical trials to assess the efficacy of ISTH0036 as an antifibrotic agent that inhibits glaucoma pathophysiological mechanisms by selectively suppressing TGF-β2 [321].

Other TGF-β antisense oligonucleotides, including TASO-001, STP705, and TRK250, are under evaluation in clinical trials (Table 2).

### Vaccine-based therapy

#### Belagenpumatucel-L

Belagenpumatucel-L (Lucanix™) is a nonviral gene-modified allogeneic whole tumor cell vaccine expressing the antisense strand of the TGF-β2 gene. This approach is well tolerated in a phase II study of Belgel-L for non-small cell lung cancer [322]. During therapy, baseline circulating tumor cells are associated with overall survival [14]. In a phase III trial for non-small cell lung cancer, there is a difference in overall and progression-free survival between the belagenpumatucel-L group and the placebo group [323]. As the phase III trial of belagenpumatucel-L for stage III/IV non-small cell lung cancer does not meet the primary end point, further studies are needed to select patients who may benefit from this vaccine [324].

#### Vigil

Vigil (Gemogenovatucel-T, FANG™, IND14205) is an autologous compound consisting of a plasmid encoding granulocyte-macrophage colony-stimulating factor (GM-CSF) and a bifunctional short hairpin RNAi (bishRNAi) targeting furin convertase, leading to downregulation of TGF-β1 and β2. There is a phase I study for advanced cancer, and this trial shows that Vigil is successful in 42 of 46 patients, of whom 27 receive over one vaccine. There are no serious adverse events after treatment [325]. The three-year follow-up of Vigil in 12 patients with metastatic advanced Ewing’s sarcoma reveals a one-year survival of 73% for Vigil-treated patients compared to 23% in the placebo group. The overall survival is 17.2 months between the Vigil (median overall survival of 731 days) and placebo groups (median overall survival of 207 days) [326].

In advanced ovarian cancer, an induction of the circulating activated T-cell population is observed in the Vigil group [327]. In the phase IIB (VITAL) trial for stage III/IV ovarian cancer, utilizing Vigil as maintenance immunotherapy is well tolerated. However, the primary endpoint is not met after the treatment (NCT02346747) [328]. However, Vigil is demonstrated to have clinical benefit for ovarian cancer with homologous recombination proficient. The recurrence-free and overall survival are improved in the Vigil group compared to the placebo group [329]. The three-year follow-up of Vigil for patients with homologous recombination-proficient ovarian cancer still shows durable activity in both recurrence-free and overall survival [330]. The gene expression profile suggests that Vigil’s overall survival benefit is correlated with elevated expression of MHC-II and positive IFN-γ ELISPOT in patients with recurrent ovarian cancer [331]. When combined with atezolizumab in relapsed ovarian cancer patients, the median overall survival is not reached. However, patients harboring BRCA^wt^ suggest an improved overall survival benefit. Thus, a continued investigation of combination therapy with Vigil-1st and atezolizumab is needed for patients with BRCA^wt^ [332, 333].

## Conclusions and perspectives

The multifunctional cytokine TGF-β regulates inflammatory progression, differentiation, proliferation, and wound healing during homeostasis. Dysregulated TGF-β promotes EMT and immunosuppression during tumorigenesis and fibrosis. Therefore, there is increasing interest in targeting TGF-β signals. In addition, TGF-β-targeted therapies, including neutralizing antibodies and TGF-β ligand traps for ligand elimination, small-molecule receptor kinase inhibitors, ASOs and vaccine-based therapy, have achieved comparable results in preclinical trials to treat tumors, fibrosis, and other diseases. However, few of these anti-TGF-β compounds are in phase III clinical trials because of the different roles of TGF-β in different cancer stages and the poor stability and side effects of anti-TGF-β drugs [3].

The role of TGF-β in tumorigenesis and progression is different and complex. Multiple types of research indicate that TGF-β becomes a tumor suppressor at an early stage. In contrast, at a late stage, overexpressed TGF-β promotes the formation of EMT, TME, immunosuppression, and CAFs. It is difficult but essential to determine whether a patient’s TGF-β is a promotor or a suppressor. More research should be ongoing to identify which tumor types or fibrosis could benefit from targeting TGF-β therapies. In addition, combination strategies could also solve cardiovascular adverse effects [334], poor stability in vivo [335], and some other side effects promoting poor therapeutics. In conclusion, progress in detecting the universal mechanisms of TGF-β in specific tumor subtypes and diverse stages of cancer, as well as other diseases, and exploring appropriate combination dosing regimens to reduce side effects are essential and prospective.

## Data Availability

The materials supporting our conclusion of this review are included within the article.

## Abbreviations

TGF-β: Transforming growth factor beta
ECM: Extracellular matrix
LAP: Latency-associated peptide
GARP: Glycoprotein-A repetitions predominant
LRRC32: Leucine-rich repeat containing 32
EMT: Epithelial-to-mesenchymal transition
JNK: c-Jun N-terminal kinase
NF-κB: Nuclear factor kappa-B
TRAF4/6: Tumor necrosis factor-associated factor 4/6
ERK: Extracellular signal regulated kinase
RREB1: RAS-responsive element-binding protein 1
PDGF: Platelet-derived growth factor
TME: Tumor microenvironment
TAMs: Tumor-associated macrophages
MDSCs: Myeloid-derived suppressor cells
DCs: Dendritic cells
CAFs: Cancer-associated fibroblasts
Tregs: Regulatory T cells
NK cells: Natural killer cells
IFN-γ: Interferon-γ
IL-2: Interleukin-2
PD-1: Programmed death 1
mTOR: Mammalian target of rapamycin
CTGF/CCN2: Connective tissue growth factor
BMP-7: Bone morphogenic protein-7
TIF1gamma: Transcriptional intermediary factor 1γ
HPV: Human papillomavirus
PD-L1: Programmed cell death protein ligand 1
ASOs: Antisense oligonucleotides
GM-CSF: Granulocyte-macrophage colony-stimulating factor
